# Synaptic Function and Dysfunction in Lysosomal Storage Diseases

**DOI:** 10.3389/fncel.2021.619777

**Published:** 2021-03-04

**Authors:** Rima Rebiai, Maria I. Givogri, Swetha Gowrishankar, Stephania M. Cologna, Simon T. Alford, Ernesto R. Bongarzone

**Affiliations:** ^1^Department of Anatomy and Cell Biology, College of Medicine, The University of Illinois at Chicago, Chicago, IL, United States; ^2^Department of Chemistry, College of Liberal Arts and Sciences, The University of Illinois at Chicago, Chicago, IL, United States

**Keywords:** lysosomes, synapses, glutamate receptors, GABA, sphingolipids, cholesterol, LTP, LTD

## Abstract

Lysosomal storage diseases (LSDs) with neurological involvement are inherited genetic diseases of the metabolism characterized by lysosomal dysfunction and the accumulation of undegraded substrates altering glial and neuronal function. Often, patients with neurological manifestations present with damage to the gray and white matter and irreversible neuronal decline. The use of animal models of LSDs has greatly facilitated studying and identifying potential mechanisms of neuronal dysfunction, including alterations in availability and function of synaptic proteins, modifications of membrane structure, deficits in docking, exocytosis, recycling of synaptic vesicles, and inflammation-mediated remodeling of synapses. Although some extrapolations from findings in adult-onset conditions such as Alzheimer’s disease or Parkinson’s disease have been reported, the pathogenetic mechanisms underpinning cognitive deficits in LSDs are still largely unclear. Without being fully inclusive, the goal of this mini-review is to present a discussion on possible mechanisms leading to synaptic dysfunction in LSDs.

## Introduction

Lysosomal storage diseases (LSDs) are characterized by the accumulation of biological macromolecules in the lysosomes, resulting in the formation of large intracellular deposits that affect cellular function. LSDs are inherited diseases of the metabolism caused by genetic deficiencies of proteins and enzymes involved in the biogenesis and/or degradative function of lysosomes (Table 1; Greiner-Tollersrud and Berg, 2000–2013). Lysosomal dysfunction in LSDs may impact the degradation of lipids, proteoglycans, proteins, and also those proteins involved in lysosomal trafficking and transporters (Table 1; Greiner-Tollersrud and Berg, 2000–2013). Most often, LSDs present infantile symptoms, although juvenile and late-onset forms also exist. Manifestations in early-onset neurological LSDs may include hearing loss, seizures, neuromotor regression, demyelination, intellectual disability, and developmental delay. Late-onset cases may also develop depression, psychosis, and dementia. About two-thirds of the patients affected with LSDs experience neurological deficits, and thus, a correlation between synaptic failure and cognitive decline may apply to these LSD patients (Morrison and Baxter, 2012). Although several studies have described morphological and functional changes in neurons, synapses (Lepeta et al., 2016) and electrophysiological deficits in LSDs (Zoghbi and Bear, 2012; Calabrese et al., 2016; He et al., 2018), important questions on linking lysosomal storage defects with changes in structure and function of synapses remain largely unaddressed.

**Table 1:** Examples of lysosomal storage diseases (LSDs) where cognitive defects were observed.

Disease	Defective gene	Signs and symptoms
**Defects in glycoprotein degradation**
Mucolipidosis I, Sialidosis	α-Sialidase	Cherry-red macules in the eyes. Coarse facial features. Skeletal malformation and mental delay.
Galactosialidosis	Cathepsin A	Difficulty coordinating movements. Muscle twitches. Seizures. Visual loss. Dark red spots on the skin.
α-Mannosidosis	α-Mannosidase	Intellectual disability, hearing loss, ataxia, skeletal abnormalities, and coarse facial features.
β-Mannosidosis	β-Mannosidase	Respiratory infections. Swallowing difficulties. Poor muscle tone. Hearing loss. Speech impairment.
Aspartylglucosa-minuria	Glycosylasparaginase	Recurrent infections. Diarrhea. Gradual coarsening of facial features. An enlarged tongue and liver.
Fucosidosis	α-Fucosidase	Seizures. Abnormal bone development and muscle stiffness. Dark red spots on the skin.
Schindler	α-N-Acetylglucosaminidase	Redness and development of clusters of wart-like discolorations on the skin. Intellectual impairment.
**Defects in glycolipid degradation**
GM1 gangliosidosis MPS IVB	β-Galactosidase	Poor muscle tone. Enlargement of liver and spleen. Skeletal abnormalities. Seizures. Visual impairment.
GM2 gangliosidosis Tay-Sachs/Sandhoff	β-Hexosaminidase (α) β-Hexosaminidase (β) GM2 activator protein	Feeding problems. Cherry red spots in the backs of the eyes. Severe and fatal mental and physical deterioration.
Gaucher disease	Glucocerebrosidase Saposin C	Spleen/liver enlargement. Blood and bone issues. Mental and motor problems.
Globoid cell leukodystrophy	β-Galactosylceramidase	Muscle weakness. Feeding difficulties. Severe seizures and fevers. Vision and hearing loss.
Metachromatic leukodystrophy	Arylsulfatase A Saposin B	Walking difficulties, marked spasticity, seizures, and profound mental retardation.
Multiple sulfatase deficiency	Formyl-Glycin generating enzyme	Abnormality of peripheral nerve conduction. Developmental delay. Enlarged liver.
Fabry	α-Galactosidase A	Severe burning pains in hands and feet. Distinctive skin rash on the legs. Kidney & heart failure. Strokes.
**Defects in glycogen degradation**
Pompe	α-Glucosidase	Heart enlargement and heart failure in infants. Respiratory problems and severe muscle weakness.
**Defects in sphingomyelin degradation**
Niemann Pick type A and B	Acid sphingomyelinase	Organ enlargement. Lung dysfunction and central nervous system damage for certain subtypes.
Farber lipogranulomatosis	Acid ceramidase	A hoarse voice & weak cry, small lumps of fat under the skin and tissues, swollen and painful joints.
**Defects in triglycerides and cholesteryls esters degradation**
Wolman/cholesteryl ester storage disease	Acid lipase	Anemia, vomiting and diarrhea. Developmental delay. Poor weight gain. Low muscle tone.
**Defects in protein degradation**
Pycnodystostosis	Cathepsin K	A large head and high forehead. Undeveloped facial bones. Short fingers and toes. Dental abnormalities.
Ceroid lipofuscinosis 2 Ceroid lipofuscinosis 1	Tripeptidyl peptidase Palmitoyl-protein Thioesterase	Abnormally increased muscle tone or spasm. Vision problems. Dementia. Lack of muscle coordination. Intellectual disability. Loss of speech. Seizures.
**Defects in lysosomal transporters**
Cystinosis	Cystinosin (cystin transport)	Impaired kidney function. Increased sensitivity to light, and marked growth retardation.
Salla disease	Sialin (sialic acid transport)	Intellectual disability and seizures. Problems with movement and balance. Muscle tension.
**Defects in lysosomal trafficking proteins**
Mucolipidosis III (I-cell)	Phosphotransferase γ-subunit	Abnormal skeletal development. Delayed motor skills. Enlargement of liver, spleen, & heart valves.
Mucolipidosis IV	Mucolipin-1(cation channel)	Intellectual disability. Diminished muscle tone. Clouding (opacity) of the clear portion of the eyes.
Danon	LAMP-2	Muscle problems. Delayed motor skills. Intellectual disability. Shortness of breath and visual complaints.
Niemann Pick type C	NPC1	Organ enlargement. Lung dysfunction and central nervous system damage for certain subtypes.
Batten disease Ceroid lipofuscinosis 3, 6, 8	CLN3- CLN 6- CLN 8	Vision failure. Recurrent seizures. Neurological disturbances. Muscle rigidity. Impaired speech.
Chediak-Higashi	LYST	Sensitivity to light. Blond or light brown hair with a silvery tint. Damaged immune and nervous systems.
Griscelli Type 1 Griscelli Type 2 Griscelli Type 3	MYOV RAB27A Melanophilin	Distinctive skin and hair coloring. Weak muscle tone. Delayed development. Intellectual disability. Vision issues. Liver enlargement. Immunodeficiency.
Hermansky Pudliak 2	AP3 β-subunit HOPS complex: VPS11, VPS16, VPS18, VPS39, VPS41 and VPS33A BLOC complexes 1, 2, & 3	Dysfunction of blood platelets leading to prolonged bleeding. Lack of skin, hair, and eye pigmentation.
**Defects in glycosaminoglycan degradation**
MPS II (Hunter) MPS 1 (Hurler, Scheie) MPS IVA (Morquio A) MPS IX MPS IIIa (Sanfilippo A) MPS IIIc (Sanfilippo C) MPS IIIb (Sanfilippo B) MPS IIId (Sanfilippo D) MPS VII (Sly) MPS VI	Iduronate sulfatase α-Iduronidase Galactose 6-sulfatase Hyaluronidase Heparan N-sulfatase Acetyl-CoA transferase N-acetyl-glucosaminidase N-acetyl glucosamine 6-sulfatase *β*-glucuronidase N-Acetylgalactosamine 4-sulfatase	Bone and joint deformity as well as interference with normal growth. Weight-bearing joints.

## Synapse Pathology in Neurological LSD

Numerous studies indicate that synaptic plasticity underlies the neurobiological basis of higher cognitive function. Dysfunction in synaptic transmission (synaptic failure and/or synaptic death) undermines mechanisms of synaptic plasticity, leading to loss of synaptic function, which may further facilitate a neurological decline in multiple pathological conditions (Lepeta et al., 2016). Spine morphology is tightly linked to synapse function and has been used often as a readout of synaptic deterioration (Holtmaat and Svoboda, 2009; Bosch and Hayashi, 2012). Progressive atrophy in spines in Alzheimer’s disease (AD) and progressive neuronal dysfunction in Parkinson’s disease (PD) are possibly good examples of this. AD is characterized by the loss of synapses and neurons in the cerebral cortex and hippocampus, as well as the formation of Aβ plaques and neurofibrillary lesions. This progressive loss of synaptic structure is believed to impair synaptic plasticity and memory and lead to cognitive decline in AD patients (Hsieh et al., 2006). Interestingly, in addition to plaques, AD has also evidence of lysosomal storage, suggesting a potential pathogenic contribution of lysosomes to AD (Orr and Oddo, 2013). Of importance, genetic associations between LSDs and adult-onset conditions such as AD and PD have been recently identified. For example, mutations in the gene GBA (encoding for glucosylceramidase, deficient in Gaucher’s disease, GD) are genetic risk factors associated with PD (Barkhuizen et al., 2016; Gan-Or et al., 2018; Sidransky et al., 2009). Additionally, SMPD1 (acid sphingomyelinase, deficient in Niemann Pick type A, B), ASAH1 (acid ceramidase, deficient in Farber’s disease and spinal muscular atrophy with progressive myoclonic epilepsy), ARSA [arylsulfatase A, deficient in metachromatic leukodystrophy (MLD)] and GALC [lysosomal galactosylceramidase, deficient in Krabbe’s disease (KD)] are also associated with vulnerability to develop adult onset neurodegenerative disorders, particularly PD (Smolders and Van Broeckhoven, 2020). These studies underline the possibility that mechanisms involved in synaptic failure in LSDs may also be playing an active role in other neurodegenerative diseases.

Analogous structural studies have started to find abnormalities in some LSDs, providing further evidence of synaptic dysfunction. For example, abnormal spine morphology (immature and reduced dendritic spines) has been reported in animal models of LSDs including mucopolysaccharidosis type IIIC or Sanfilippo disease type C and Tay-Sachs’ disease (Dwyer et al., 2017; Sambri et al., 2017). Additionally, these studies showed an abnormal distribution of several pre- and post-synaptic proteins, disorganized microtubule networks, reduced postsynaptic densities, lower synaptic vesicles, and alterations in frequency and amplitude of miniature EPSCs and IPSCs (Dwyer et al., 2017; Sambri et al., 2017). Studies in *Drosophila* mutants lacking neuronal glucosylceramidase (modeling for GD) have also found major synaptic loss and neurodegeneration (Kinghorn et al., 2017). An Npc1−/− mouse model for Niemann-Pick type C disease (NPC), which results from defects in cholesterol transport, develops significant alteration in presynaptic structure and function with important neuronal loss (Wang et al., 2010; Peake et al., 2011) and impairment of long-term synaptic potentiation (LTP; D’Arcangelo et al., 2016).

**Figure 1:**
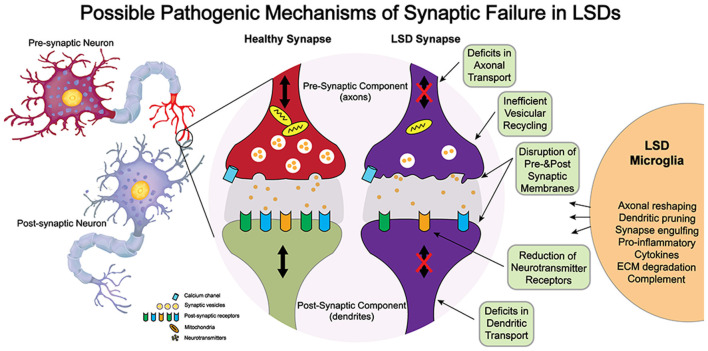
Potential mechanisms of neuropathogenesis affecting synaptic structure and function in lysosomal storage diseases (LSDs).

Although these structural studies point to synaptic dysfunction in some LSDs, much less is known on how a given lysosomal deficiency triggers these changes. Because synapses are highly complex structures with multiple components, it is unlikely that synaptic dysfunction in LSDs involves direct genetic defects of synaptic components, which tend to be embryologically lethal, but rather, are elicited by indirect defects arising from lysosomal dysfunction. Among others, synaptic efficacy may be impacted by alterations in the architecture of pre- and/or post-synaptic membranes; slowed vesicular transport reducing delivery of mitochondria and other components needed for synaptic vesicle formation; inefficient formation of SNARE complexes; and altered lysosomal/autophagosomal activity (Virmani et al., 2005; Xu et al., 2010; Button and Luo, 2017; Button et al., 2017; Sharma and Lindau, 2017). Furthermore, the exocytosis and recycling of synaptic vesicles (Rangaraju et al., 2014) and the speed and specificity of protein sorting in presynaptic active zones (Bonanomi et al., 2006) and post-synaptic structures—critical for learning (Malinow and Malenka, 2002) and homeostasis (Pérez-Otaño and Ehlers, 2005)—impose a very heavy metabolic burden on synapses. Hence, an interplay of factors such as accumulation of undegraded substrates within the synaptic endo-lysosomal pathway, altered lysosomal pH and membrane, endoplasmic reticulum stress, unfolded protein response activation, deformation of synaptic membrane architecture (e.g., shedding and invagination), autophagy impairment, and inflammation (Kim et al., 2006; Tessitore et al., 2009; Weinert et al., 2010; Hawkins-Salsbury et al., 2013; Lim et al., 2015; Karch et al., 2017; Zucca et al., 2018; Gabandé-Rodríguez et al., 2019) are more likely to contribute to synaptic disease in neurological LSDs. Figure 1 illustrates some of the possible pathways contributing to synaptic failure in LSDs. For most LSDs, the initial triggers of synaptic dysfunction are still unknown. Below, we discuss the involvement of lipids, dysregulation of synaptic components, and the role of inflammation.

## Defects in Synaptic Function Mediated by Lipids

Because synapses are so closely dependent on membrane dynamics (Ceccarelli et al., 1973; Heuser and Reese, 1973), pathological conditions altering the recycling of lipid components during synaptic vesicle release and recycle are expected to impact the architecture of affected synapses. In sphingolipidoses such as KD and MLD, lysosomal defects lead to the progressive accumulation of undegraded sphingolipids in multiple membrane compartments. The build-up of psychosine (KD) and sulfatides (MLD) in lipid rafts of brain membranes provided an initial rationale to understand the link between membrane deformation and synaptic dysfunction in these LSDs (White et al., 2009; Moyano et al., 2014; Sural-Fehr et al., 2019). Psychosine is the main undegraded sphingolipid accumulated in KD caused by the deficiency of galactosylceramidase (Table 1; White et al., 2009). At high levels, psychosine becomes cytotoxic, particularly to oligodendrocytes, resulting in extensive demyelination observed in the KD brain (Suzuki, 1998). However, psychosine accumulates and disrupts the architecture of most brain membranes by altering the composition and dynamics of lipid rafts (White et al., 2009; Hawkins-Salsbury et al., 2013) and promoting membrane shedding (D’Auria et al., 2017). Lipid rafts are generally considered subdomains in the membrane where the association of sphingolipids and cholesterol facilitates the interaction with coupled raft-proteins. Some of these proteins are receptors, G-proteins, and enzymes which regulate a myriad of different signaling pathways (Bieberich, 2018). Thus, we could elaborate that the direct effect of the genetic trait in KD (i.e., the deficiency of a lysosomal enzyme in charge of degrading sphingolipids primarily impacting on myelin membranes) also leads to the accumulation of psychosine in other cell membranes, which indirectly induces an array of secondary pathogenic events. Depending on the cellular context where this occurs, distinct pathogenic mechanisms may be activated. For example, in neurons psychosine induces the dephosphorylation of neurofilaments by deregulation of two serine/threonine phosphatases (PP1 and PP2A) effecting neuronal cytoskeleton (Cantuti-Castelvetri et al., 2012). Psychosine promotes the abnormal activation of GSK3β and the reduction of fast axonal transport mechanisms (Cantuti Castelvetri et al., 2013), indirectly reducing the speed and efficiency of synaptic protein delivery. These changes are thought to be facilitated by the release of dephosphorylating activities (e.g., PP1 and PP2A) from the membrane, in response to increased local concentrations of psychosine. Because changes in pre- and post-synaptic proteins, their assembly, and axonal/dendritic transport are associated with cognitive decline (Attems and Jellinger, 2018), psychosine might increase synaptic vulnerability by inducing membrane disorganization. Additionally, psychosine binds and facilitates the abnormal aggregation of α-synuclein in neurons (Abdelkarim et al., 2018), suggesting additional and more direct involvement in neuronal pathology in KD. From our experience with psychosine, we could expect that some common mechanisms involving membrane deformation apply to other lipidoses such as MLD, GD, and NPC. While there is clear evidence of a cognitive decline in all age-related MLD variants (Hyde et al., 1992; Gieselmann et al., 1998), most reports support the hypothesis that neuronal dysfunction is primarily owed to the accumulation of sulfatides in oligodendrocytes, and the indirect damage to axon integrity and maintenance caused by demyelination (Ishibashi et al., 2002; Honke, 2013; McGonigal et al., 2019). In other lysosomal sphingolipidoses such as gangliosidoses and GD, similar mechanistic pathways may also contribute to triggering neuronal deficits (Lee et al., 2012). Thus, a growing body of evidence indicates that changes in lipid homeostasis seen in various lipidoses also are capable to trigger structural changes in synaptic membranes and function (Egawa et al., 2016; Díaz et al., 2018).

## Potential Defects in Pre- and Post-Synaptic Components in LSDs

Systematic studies of pre- and post-synaptic components are largely missing and at best fragmented and incomplete for most LSDs. We can infer potential mechanisms affecting these components in LSDs from a larger and deeper understanding of synaptic components, structure, and function from formal studies in normal conditions. Presynaptic SNARE proteins (e.g., syntaxin-1, SNAP25, and VAMP2) represent the core machinery for synaptic vesicle fusion (Sutton et al., 1998) and are crucial for synaptic transmission (Hayashi et al., 1994) and are predictors of cognitive function (Ramos-Miguel et al., 2017). Analysis of these pre-synaptic proteins suggests that the level of their functional interactions is associated with better cognition, and less decline over time (Ramos-Miguel et al., 2017). Failure to properly form SNARE complexes interfere significantly with synaptic transmission. Additionally, the regulation of SNARE function is complex and offers several points where dysregulation may occur, particularly because of association with membrane lipids. Of relevance, SNAREs have various affinities for sphingolipid and sterol-rich lipid raft domains among other lipids (phospholipids, phosphoinositides), implying the alterations in lipid homeostasis in LSD also influence SNARE functionality (Salaün et al., 2005; Lam et al., 2008; Tong et al., 2009). Moreover, phosphorylation of SNARE proteins such as SNAP-25 by protein kinases plays a key role in the exocytosis of synaptic vesicles (Gao et al., 2016). The formation of functional SNARE complexes is greatly inhibited by PKA phosphorylation of SNAP-25 at Thr 138, while it is promoted by PKC phosphorylation at Ser 187 (Gao et al., 2016). Thus, abnormal signaling regulating SNARE components may impact trafficking, docking, and release of vesicles in the axonal terminal.

Although dysregulation of signaling pathways in various cellular contexts has been described in many LSDs (Seranova et al., 2017), less is known on how these changes impact SNARE function. For example, large aggregates of presynaptic proteins including VAMP2, SNAP25, and synaptophysin were observed within axonal spheroids in several brain areas in the mouse model for NPC (Pressey et al., 2012). These findings suggest that deficient transport of cholesterol, which biochemically characterizes NPC, negatively impacts the translocation/assembly of SNARE (Pressey et al., 2012) components, but other mechanisms might also be involved. For example, in mice with MPS IIIA, reduced levels of SNAP25 and VAMP2 proteins parallel decreases in synaptic vesicles, abnormal vacuoles, and enlarged mitochondria in axonal termini (Sambri et al., 2017), but surprisingly, without defects in the expression of SNAP25 and VAMP2 mRNAs. This underlines the possibility that mutant neurons might activate abnormal degradative pathways rather than impaired gene expression, impacting the availability of functional SNARE components for proper action at the presynaptic terminal (Sambri et al., 2017). This mechanism may be a consequence of dysfunctional lysosomal-autophagosomal activities, a pathogenic mechanism observed in most LSDs (Lieberman et al., 2012).

Defective transport of SNARE components in LSDs might be elicited by the loss of other synaptic components such as α-synuclein, which is implicated in neuronal loss in PD and Lewy Body dementia, and cysteine string protein α (CSPα). These two abundant presynaptic proteins act as chaperones and contribute to the SNARE complex formation at synaptic terminals (Chandra et al., 2005; Burré et al., 2010; Burgoyne and Morgan, 2011; Sharma et al., 2011). Defects in axonal transport have been described in several conditions and recently, associated with some LSDs. In KD, defects in axonal transport were first described by Cantuti Castelvetri et al. (2013). These authors found that both anterograde and retrograde transport rates are affected by psychosine. Slower transport may reduce SNARE proteins’ availability, recycling of synaptic vesicles, and translocation of mitochondria. These results were further supported by measurements of slowed retrograde transport of synaptophysin-positive vesicles in Krabbe neurons, indicating that the early steps of endocytosis and retrograde transport of endocytic and synaptic vesicles can also be impaired (Teixeira et al., 2014). Defects in the availability of functional pre-synaptic components have also been suggested for other LSDs including MPSI and IIIA (Wilkinson et al., 2012; Baldo et al., 2015), MPS IIIB (Vitry et al., 2009), MPS VII (Bayó-Puxan et al., 2018), CLN6 (Kanninen et al., 2013), and CLN5 (Amorim et al., 2015).

Likewise, defects in post-synaptic components might contribute to overall synaptic failure in LSDs. For example, several neurotransmitter receptors must be localized in lipid rafts for efficient nerve transmission (Allen et al., 2007). Regardless of their different brain localization, AMPA receptors (particularly GluR1, GluR2/3, and GluR4) along with GABA receptor A, acetylcholine receptors, and NMDA receptors (NR1, NR2A, and NR2B), are most often associated with lipid rafts. The movement between the raft and non-raft domains in the synaptic membrane is regulated by several signaling mechanisms, which can be targeted for dysregulation in LSDs. For instance, studies on KD show that psychosine facilitates the redistribution of raft-associated proteins such as Flotillin-2 and Caveolin-1 and leads to a generalized and likely irreversible disruption of membranes *via* shedding (White et al., 2009; D’Auria et al., 2017). Thus, we can hypothesize that in KD, psychosine-induced shedding severely changes the local concentration of key receptors and other proteins needed for optimal nerve transmission.

Similarly, we could envisage that alterations in postsynaptic raft composition and/or activity of associated signaling pathways may impact the recruitment of neurotransmitter receptors in other LSDs. For instance, the activation of CaMKII facilitates the trafficking of AMPAR to the dendritic rafts. CaMKII phosphorylates the GluA1 AMPA subunit at Ser831 (Kristensen et al., 2011) or, indirectly, through the activation of the Ras/MAPK pathway (Henley and Wilkinson, 2013), and genetic defects in the Ras-MAPK pathway are associated with deficient plasticity (Cesarini et al., 2009; Lisman et al., 2012). As in presynaptic terminals, the localization of receptors requires exocytic and endocytic processes involving SNARE complex-dependent vesicle transport, and aberrant receptors (e.g., AMPA) trafficking has been associated with impaired synaptic plasticity and cognitive deficits (Henley and Wilkinson, 2013; Chater and Goda, 2014). As stated before, little is known on neurotransmitter receptors trafficking, assembly, and regulation in the context of LSDs. Studies in a mouse model of CLN1 showed deficient developmental switch in NMDA receptors from GluN2B to GluN2A and reduced interactions between SAP-102 with GluN2B and PSD-95 with GluN2A, associated with early synaptic impairments (Koster et al., 2019). Studies in the MPS IIIA mouse have shown that accumulation of heparan sulfate in the cerebral cortex correlates with altered levels of AMPA receptor GluA2 and enhanced puncta for postsynaptic density 95 (PSD-95) in the somatosensory cortex, providing a potential link to study defects in synaptic neurotransmission (Dwyer et al., 2017). Other studies have also shown significant reductions in Homer-1, a protein enriched in the postsynaptic density of excitatory synapses in the brain of MPS I, IIIA, and IIIB (Wilkinson et al., 2012). Defects in receptor localization and interaction at the postsynaptic density likely facilitate an impaired receptor conductance state. For instance, the kinetics of the NMDA excitatory postsynaptic currents (EPSCs) showed longer activation times of GluN2B receptor (Koster et al., 2019), indicating impairment in NMDA insertion/removal from the synaptic membrane affecting both synaptic homeostasis and plasticity. Together, these few studies in LSDs underline that deregulation in several post-synaptic proteins may also be common traits affecting synaptic function in LSDs (Wilkinson et al., 2012). Undoubtedly, more studies are needed to determine whether common or disease-specific pathogenic mechanisms are present in other neurological LSDs.

## Potential Defects in Synaptic Transmission and Strength in LSD

During exocytosis, synaptic vesicles fuse with the plasma membrane at active zones to release neurotransmitters into the synaptic cleft. These vesicles will then be recycled to make new ones *via* endocytosis (Soykan et al., 2016). Endocytosis and exocytosis at the synaptic terminals is an exciting area of study, which might be of crucial relevance in many LSDs (Xu et al., 2010). For instance, evoked vesicle exocytosis was impaired in hippocampal neuronal cultures of NPC1 mice (Xu et al., 2010) and reductions in docked synaptic vesicles in cortical neurons from CLN1 mice have been described (Virmani et al., 2005). Decreased numbers of early endosomes expressing Ras-related protein 5 were also detected in dorsal root ganglion neurons from the twitcher mouse, a model for KD (Teixeira et al., 2014). Similarly, MPS IIIA neurons develop a phenotype characterized by reduced synaptic vesicles and decreased exocytosis rate (Sambri et al., 2017).

Because the rate of synaptic vesicle exocytosis/endocytosis directly controls neurotransmission release, deficits in either process may affect synaptic strength and functions. Synaptic strength is highly influenced by AMPA (Anggono and Huganir, 2012) and NMDA receptors (Bliss and Collingridge, 1993). As discussed before, localization of AMPARs within lipid rafts at the post-synaptic membrane, which is regulated by the activity of NMDA receptors (Kristensen et al., 2011) impacts synaptic strength and can elicit significant changes in LTP and LTD. A decrease in synaptic strength during LTD implies the removal of receptors by endocytosis and sorting to degradative organelles (i.e., lysosomes) to reduce the number of available AMPA receptors at the plasma membrane. Conversely, LTP involves increasing the number and recycling of AMPA receptors to the plasma membrane (Liao et al., 1995; Qin et al., 2005). During LTD, NMDA receptor-dependent internalization of AMPA receptors involves dephosphorylation at S845 of GluA1 (Parkinson and Hanley, 2018) and phosphorylation of GluA2 at S880 (Jurado, 2018), whereas the recycling of AMPA receptors during LTP requires the phosphorylation of GluA1 at S845 and S831 (Parkinson and Hanley, 2018). We speculate that alterations in membrane composition owed to altered metabolism of lipids and other lysosomal substrates impact on the recycling and availability of receptors affecting LTD/LTP and contribute to some of the neurobehavioral manifestations observed in patients with LSDs. For example, synaptic vesicles in both excitatory and inhibitory synapses are reduced in neurons from the Npc1−/− mouse, effecting the readily released pool of vesicles (Xu et al., 2010) and reducing hippocampal LTP in NPC1 mice (Võikar et al., 2002). This could help not only to understand distinct patterns of neurobehavioral symptoms in the NPC1 patients (Sévin et al., 2007) but also to illustrate possible avenues of research of cognitive function in other LSDs.

## Inflammation as A Contributor to Synaptic Failure in LSDs

As discussed above, the efficacy of synaptic transmission can be altered at multiple levels, affecting the availability of pre- and post-synaptic components, the recycling of vesicles, and the number of neurotransmitter receptors. Activation of inflammatory responses and death-mediated pathways are also relevant mechanisms by which to modify synaptic function. For example, resting microglia are critical immune surveillance cells in charge of monitoring neuronal and glial functions in the brain and play a fundamental role in shaping synapses and synaptic plasticity during development and normal physiological activity of the nervous system (Morris et al., 2013). Expectedly, lysosomal function plays an important role in inflammation and immunity. For example, bioactive sphingolipids ceramides and sphingosine-1-phosphate are known to modulate the activation of inflammatory responses (Scheiblich et al., 2017; Sapkota et al., 2019). Glucosylsphingosine, which accumulates in GD, promotes the activation of macrophages and the release of multiple cytokines, such as chitotriosidase, TNF-α, and IL-1β (Pandey et al., 2014). During inflammatory events, microglial cells alter their function to attempt restoring homeostasis, which includes secretion of cytokines, endocytosis, and engulfment of debris. These inflammatory-related functions may drastically impact synaptic function by the secretion of factors that modify LTP/LTD (Zhou et al., 2019), degradation of extracellular matrix components inherent to the synaptic cleft (Tremblay et al., 2010; Nguyen et al., 2020), engulfment of synaptic terminals (Perry and O’Connor, 2010), and reshaping of dendrites (Cangalaya et al., 2020). An important arm of the inflammatory response in the brain is the infiltration of complement-related proteins, which have been shown to actively participate in synapse elimination (Stevens et al., 2007). This is yet another area of LSDs with minimal and fragmented understanding.

In addition to inflammation, caspases are proteins that while serving key functions in programmed cell death (Kumar, 2007), also participate in the shaping of synapses (Jonas, 2006). In particular, caspases 3, 6, and 9 appear to have key roles in dendritic pruning and axonal reshaping during learning and memory (Mattson and Duan, 1999; Forrest et al., 2013). For example, AMPA receptor subunits are known caspase substrates (Lu et al., 2002). Although the activity of caspases and other cell-related molecules is crucial during synaptic formation and maturation, their overactivation has been linked with several late-onset neurodegenerative disorders with synaptic failure (Su et al., 2001; Martin et al., 2002). Little is known about the involvement of caspases in synaptic dysfunction in LSDs. Cantuti et al reported abnormal expression of caspase 3 in presynaptic terminals of the twitcher mouse, which underlines the possibility that exacerbated degradation of synaptic components by caspase activities impact on nerve transmission (Cantuti-Castelvetri et al., 2015). Considering that inflammation of the nerve tissue is an integral part of the cellular response in most neurological LSDs (Moskot et al., 2018), the activation of microglia, inflammation, and cell death pathways are likely to influence synaptic function in these diseases and contribute to some of the neurobehavioral manifestations observed in patients.

## Concluding Remarks

Pathogenic mechanisms in most LSDs are multifactorial. Understanding those pathogenic mechanisms underlying synaptic failure in LSDs is critical not only to provide deeper integration and full interpretation of common vs. specific mechanisms at play in these diseases but also to enable further evaluation of short- and long-term responses of synaptic function to available treatments. Most LSDs lack a cure, but many are treated with enzyme correction approaches such as enzyme replacement therapy or hematopoietic transplantation (Wynn et al., 2009). Furthermore, gene therapy and small molecule (i.e., inhibitors, chaperones, etc.) interventions are also rapidly gaining relevance in treating some LSDs (Seregin and Amalfitano, 2011), and the concept of synergy from combining treatments is being seriously considered (Hawkins-Salsbury et al., 2015). Importantly, despite these advances, there is limited information on the extent to which synaptic function recovers or is protected in response to treatments and the extent and duration to which it can be sustained in patients undergoing a specific treatment. More in-depth studies into the triggering mechanisms modifying synaptic function and their response to different treatments are expected to contribute to a better understanding of pathogenesis and how to improve therapeutic safety and efficacy for LSDs.

## Author Contributions

All authors reviewed, edited, commented, and wrote the manuscript. All authors contributed to the article and approved the submitted version.

## Conflict of Interest

ERB is a consultant for E-Scape Bio, Bial, Affinia Therapeutics, Gain Therapeutics and Neurogene. The remaining authors declare that the research was conducted in the absence of any commercial or financial relationships that could be construed as a potential conflict of interest.
